# Pivotal Roles of Peroxisome Proliferator-Activated Receptors (PPARs) and Their Signal Cascade for Cellular and Whole-Body Energy Homeostasis

**DOI:** 10.3390/ijms19040949

**Published:** 2018-03-22

**Authors:** Shreekrishna Lamichane, Babita Dahal Lamichane, Sang-Mo Kwon

**Affiliations:** 1 Laboratory for Vascular Medicine and Stem Cell Biology, Medical Research Institute, Department of Physiology, School of Medicine, Pusan National University, Yangsan 50612, Korea; lamichaneshreekrishna@gmail.com (S.L.); dahalbabita2@gmail.com (B.D.L.); 2 Convergence Stem Cell Research Center, Pusan National University, Yangsan 50612, Korea; 3 Research Institute of Convergence Biomedical Science and Technology, Pusan National University Yangsan Hospital, Yangsan 50612, Korea

**Keywords:** PPARs, energy homeostasis, fatty acid oxidation, glucose-lipid metabolism

## Abstract

Peroxisome proliferator-activated receptors (PPARs), members of the nuclear receptor superfamily, are important in whole-body energy metabolism. PPARs are classified into three isoforms, namely, PPARα, β/δ, and γ. They are collectively involved in fatty acid oxidation, as well as glucose and lipid metabolism throughout the body. Importantly, the three isoforms of PPARs have complementary and distinct metabolic activities for energy balance at a cellular and whole-body level. PPARs also act with other co-regulators to maintain energy homeostasis. When endogenous ligands bind with these receptors, they regulate the transcription of genes involved in energy homeostasis. However, the exact molecular mechanism of PPARs in energy metabolism remains unclear. In this review, we summarize the importance of PPAR signals in multiple organs and focus on the pivotal roles of PPAR signals in cellular and whole-body energy homeostasis.

## 1. Introduction

Energy is essential for the survival of all living organisms, and energy metabolism describes the process of generating energy from nutrients. In humans, dietary-derived glucose and long-chain fatty acids are used as sources of energy. Energy demand in cells is fulfilled by oxidative metabolism in mitochondria. Demand and supply within cells of differing physiological states are controlled by a transcriptional regulatory network in both normal and induced cells, for example, when exercising or fasting. Peroxisome proliferator-activated receptors (PPARs) are members of a nuclear receptor superfamily within this network that regulate nutrient-dependent transcription. These receptors were first identified in the 1990s in rodents and named after their property of peroxisome proliferation [1,2,3]. In more recent work, it has become clear that PPARs also regulate gene transcription of eicosanoids and fatty acids (FAs) [4]. Moreover, PPARs have been established as a group of structurally diverse chemicals associated with transcriptional activation of the peroxisome FA β-oxidation system [5].

Similar to the other nuclear receptor family members, PPARs have a canonical domain structure. They possess an amino terminal region, which comprises a DNA binding domain and a ligand-independent transactivation domain, AF-1. At the carboxyl terminal region is a dimerization and ligand-binding domain with a ligand-dependent transactivation domain, AF-2 [6,7]. Different from other nuclear receptors, the ligand binding pocket of PPARs is unusually large and can accommodate a variety of endogenous lipids, including FAs, eicosanoids, oxidized and nitrated FAs, and derivatives of linoleic acids [8]. 

Three isoforms of PPAR, α, β/δ, and γ, have been identified and are each expressed in various tissues. PPARγ may be further classified as PPARγ-1, γ-2, and γ-3 [2]. PPARγ-2 is generated by alternative splicing and contains 28 additional amino acids at the N-terminal region compared to PPARγ-1. PPARγ-3 is a splicing variant of PPARγ-1 that gives rise to the same protein [9]. Three PPAR isoforms exhibit 80% homology and are more divergent in the ligand-binding domain, explaining their different responses to various ligands [10]. PPARs act as FA sensors to control many metabolic activities and they are involved in various biological processes, including adipogenesis, lipid metabolism, insulin sensitivity, inflammation, reproduction, and cell growth and differentiation [8,11,12]. They regulate this function upon activation of target genes by endogenous ligands. Binding of endogenous ligands to the ligand binding domain of the receptor causes a conformational change that facilitates PPARs to heterodimerize with the retinoid X receptor. This conformational change helps with binding and the release of small accessory molecules that are essential for transcription. The heterodimerized complex now assembled at PPAR response elements (PPREs) causes the transactivation of target genes of mitochondria and peroxisomes. This series of events regulates a network of proteins that are involved in systemic energy homeostasis [3,11,12].

PPARα is highly expressed in hepatocytes, enterocytes, as well as vascular and immune cell types, such as monocytes/macrophages, endothelial cells, smooth muscle cells, lymphocytes, and non-neuronal cells, such as microglia and astroglia. PPARα activates genes encoding enzymes involved in fatty acid oxidation (FAO), which include carnitine palmitoyltransferase 1 (CPT1), medium-chain acyl CoA dehydrogenase, acyl-CoA oxidase, fatty acyl-CoA synthase, FA transport proteins, and their derivatives to enter into the β-oxidation pathway [13]. In the liver, it plays a crucial role in FAO, thereby providing energy for peripheral tissues and elevating mitochondrial and peroxisomal fatty acid β-oxidation rates. PPARα is also involved in ketogenesis, by lowering plasma triglyceride levels and increasing plasma high-density lipoprotein (HDL) levels. PPARα is activated by several molecules such as long-chain unsaturated fatty acids, eicosanoids, and hypolipidemic drugs [9]. PPARγ is expressed in skeletal muscle, liver, heart, and intestine. Among the three types of PPARγ, PPARγ1 is expressed in a broad range of tissues, whereas PPARγ2 is limited to the adipose tissue. PPARγ3 is abundantly found in macrophages, large intestine, and white adipose tissue (WAT). In adipose tissue, PPARγ controls FA uptake, adipogenesis, adipokine production, lipid partitioning to fat, in addition to increasing insulin sensitivity. PPARβ/δ is expressed in skeletal muscle, adipocytes, macrophages, lungs, brain, and skin. It promotes FA metabolism and obesity resistance, improves insulin sensitivity, helps to form oxidative muscle fibers through exercise physiology, and suppresses macrophage-derived inflammation [3,6,8,12]. PPARβ/δ activators have been proposed for treating metabolic disease and are currently under clinical trials [9].

All three PPAR isotypes play essential roles in lipid and FA metabolism by directly binding to, and modulating, genes involved in fat metabolism [1]. Although they share similarities in function and mechanism of action, PPAR isotypes display important physiological and pharmacological differences. The metabolic effects of PPARβ/δ and PPARα are similar in promoting energy dissipation; in contrast, PPARγ promotes energy storage. PPARβ/δ enhances FAO in several tissues and normalizes plasma lipid levels. PPARγ and PPARβ/δ enhance insulin sensitivity, whereas PPARα is not involved in this process. PPARβ/δ-mediated glucose handling is not similar to that of PPARγ, but PPARγ and PPARβ/δ both are involved in skeletal muscle fiber type distribution, hepatic glucose metabolism, and pancreatic islet function [12]. PPARα promotes FAO under lipid catabolism, in events such as fasting, and PPARγ promotes lipogenesis during anabolism by acting on adipose tissue [4]. This review will discuss the role of PPARs in energy metabolism within various parts of the body.

## 2. PPAR Signals in Liver

Liver is the primary organ involved in whole-body energy metabolism because it can metabolize FAs and glucose. Among the three isoforms, PPARα is predominantly expressed in the liver where it regulates energy metabolism by FAO [5]. During fasting, it regulates FA uptake, ketogenesis, and β-oxidation [14]. In a previous study, it was demonstrated that FA uptake and FAO became suppressed in PPARα knockout mice. In addition to this, ketogenesis and gluconeogenesis were impaired in PPARα knockout mice. A different isotype, PPARβ/δ, has been shown to possess a different role in energy metabolism regulation in the liver. Overexpression of PPARβ/δ upregulates genes involved in energy metabolism, and deletion of PPARβ/δ reduces the expression of genes that are responsible for lipogenesis and utilization of glucose [1]. There was a significant decrease in the blood glucose level of PPARα-deficient mice after 24 h of fasting. Upregulation of TRB3 (an inhibitor of Akt/protein kinase B and a positive regulator of the cellular response to insulin) by the direct transcriptional control of PPARα has a negative effect on insulin signaling. It suggests that PPARα is important for glucose homeostasis in the liver [15]. FAO by PPARα in the liver also has an important role in ketosis, which fulfils the energy requirement in fasting [14]. 

PPARα enhances the expression of mitochondrial acyl-CoA dehydrogenase and, thus, it increases FA oxidation and acetyl-CoA enzyme production [12]. In the case of fasting, uptake and mitochondrial transport of FAs from adipose tissue is increased by PPARα by enhancing levels of mitochondrial HMG-CoA synthase, which converts acetyl-CoA to ketone bodies. PPARα modulates levels of glycoprotein CD36, which is responsible for FA uptake. PPARα regulates the enzymes involved in the degradation of straight chain FAs in the peroxisome. Hepatic enzymes, such as glycerol-3-phosphate dehydrogenase (GPDH) and glycerol kinase, which converts glycerol to glucose, are regulated by PPARα [15]. In the case of feeding, PPARα directs de novo lipogenesis to supply FAs that are stored as triglycerides and can be utilized in starvation [12].

Expression of PPARγ in the liver of mice causes liver steatosis. PPARs are considered as the target molecules of non-alcoholic fatty liver disease (NAFLD) and non-alcoholic steatohepatitis (NASH) that might cause liver cirrhosis. NASH is involved in the misregulation of PPAR signaling accompanied by PPARγ and SREBP-1c-mediated metabolic disorders. Administration of PPARγ ligand aggravates concanavalin A-induced liver injury. Abnormal stimulation of PPARα generates hepatocellular carcinoma through fatty liver [16].

## 3. PPAR Signals in Adipose Tissue

Adipose tissue is essential for energy homeostasis in the body. There are two functional types: WAT and brown adipose tissue (BAT). WAT acts as a caloric reservoir for other organs. In conditions of excess nutrition, it stores nutrients as lipids. During starvation, it releases energy through lipolysis. BAT is specialized for storage of lipids and increases energy expenditure by production of heat. Adipose tissues perform endocrinal functions, and secrete various hormones, cytokines, and metabolites called adipokines that signal for systemic energy metabolism. They regulate energy balance by obtaining signals from the central nervous system and metabolic activity in peripheral tissues [17,18,19]. PPARγ is extensively expressed in both types of adipose tissue. It is involved in the induction of genes that are essential for FA uptake and storage, as well as adipose tissue differentiation [20]. Ectopic expression of PPARγ in non-adipogenic cells converts them into adipocytes effectively [21]. Knockout of PPARγ in embryonic fibroblasts abolishes their differentiation into adipocytes [22]. A previous in vivo model has shown that PPARγ is essential for adipocyte generation and survival in animals. Heterozygous, dominant negative PPARγ mutations cause lipodystrophy in humans [8,23]. PPARα is highly expressed in BAT, but not in WAT, and it functions to regulate the expression of mitochondrial uncoupling proteins, UCP1 (Uncoupling protein 1) and PGC1α. Knockout of PPARα reduces the expression of these mitochondrial proteins under normal and cold exposure conditions. However, FA metabolism in BAT remains unaffected. When PPARα is activated in human and mouse adipocytes, it induces FAO gene expression and increases energy expenditure. PPARβ/δ is also expressed in both BAT and WAT. It plays an important role in the regulation of FAO and thermogenesis in BAT. When PPARβ/δ is ectopically expressed in adipose tissue, it dramatically induces the expression of genes involved in FAO, oxidative phosphorylation (OXPHOS), and thermogenesis. Furthermore, deletion of PPARβ/δ in BAT reduces the expression of FAO and thermogenic genes. The role of PPARβ/δ in WAT remains to be explored [1]. In rodents, BAT plays an important role in protection against obesity and obesity-associated metabolic problems. Activation of PPARγ in adipose tissue induces the expression of genes for fatty acid transport and storage as well as promotes de novo adipogenesis so that PPARγ activator thiazolidinediones (TZD) has been widely used in treatment of type II diabetes [8].

## 4. PPAR Signals in Skeletal Muscle

Skeletal muscle covers approximately 40% of the total body mass and is an important site for glycogen storage, insulin mediated glucose use, lipid metabolism, FAO, and glucose metabolism. In addition, it is also involved in the regulation of cholesterol and HDL levels. As a result, it has a significant role in insulin sensitivity and lipid metabolism. PPARβ/δ expression is dominant in skeletal muscle and it regulates gene expression involved in energy metabolism by relying on FAs as an energy source [14,24,25,26]. It regulates genes for triglyceride hydrolysis, lipid uptake, and FA oxidation, as well as activating uncoupling proteins to provide energy for OXPHOS. It also encodes mitochondrial protein CPT1 to regulate long chain FAO. PPARβ/δ activates FOXO1, a transcription factor for metabolic adaptation, and pyruvate dehydrogenase kinase 4 (PDK4), which inactivates the pyruvate dehydrogenase complex and is, therefore, a rate-limiting step in muscle carbohydrate oxidation. PDK4 acts on several genes that code for lipid efflux and energy expenditure [25]; it also upregulates fatty acid β-oxidation. Furthermore, glucose metabolism was shown to be increased in PPARβ/δ transgenic mice [24]. To control muscle FA metabolism, PPARβ activates gene transcription of lactate dehydrogenase B (LDHB), which is important for glucose oxidation, by converting glucose and lactate into pyruvate for mitochondrial oxidation [27]. 

Energy metabolism in skeletal muscle is regulated by PPARγ coactivator-1α (PGC-1α), a regulator of mitochondrial biogenesis [28], involved in the catabolic process to synthesize aerobic adenosine triphosphate (ATP). PGC-1α expression is directly activated by PPARβ/δ to regulate skeletal muscle metabolism by increasing the expression of mitochondrial proteins [29,30]. PGC-1α stimulates the expression of genes responsible for glucose and lipid metabolism, energy transfer, and muscle contractile function. Furthermore, PGC-1α knockout mice have shown defects in skeletal muscle energetics, and have decreased mitochondrial biogenesis and oxidative function [31]. In skeletal muscle, the increment in lipid oxidation and reduction of glucose utilization is conducted by the activation of PPARδ. In the nucleus, transcription factor EB (TFEB) induces the expression of genes involved in lysosomal biogenesis and lipid metabolism through PGC-1α during fasting [32].

## 5. PPAR Signals in Kidney

All three isoforms of PPARs (PPARα, PPARβ/δ, and PPARγ) are found in the kidney. PPARα is highly expressed in the renal proximal tubules and the medullary thick ascending limbs of Henle [33]. PPARγ is mainly found in the medullary collecting duct with low expression in glomeruli and proximal tubules [34]. The nuclear receptors, PPARα and PPARγ, are concerned with the control of FAs and glucose metabolism. FAs are the main source of fuel for energy production in kidney cortex tissue [33]. PPARγ alters large numbers of target genes involved in peripheral glucose and FA metabolism leading to improved insulin sensitivity and glycemic control [34]. PPARα is the master regulator of lipid metabolism by controlling the transcription of its target genes such as acyl-CoA oxidase, acyl-CoA, CPT1a, PGC1α, UCP2, and UCP3 [35]. It regulates renal FA β-oxidation [33,36], which provides the source of ATP in proximal renal tubular cells. PPARs regulate FAO and control energy homeostasis, as well as lipid and glucose metabolism by gluconeogenesis, stimulating ketone body synthesis and adipogenesis [33]. In renal proximal tubule cells, FA metabolites derived from arachidonic or linoleic acids via cyclooxygenase or lipoxygenase pathways activate PPARα. Mouse kidney cortex cells use polyunsaturated FAs as the primary source of energy production. Mitochondrial biogenesis is controlled by PPARα through OXPHOS, FA metabolism, and the tricarboxylic acid (TCA) cycle [37]. 

Moreover, the kidney has a role in energy balance because of its vast gluconeogenic enzyme activities including that of PDK4 and its contribution to glucose during fasting. Furthermore, fasting induces high levels of PGC-1α along with its regulating partners, estrogen-related receptors (ERRs) in the kidney, which are involved in the TCA cycle and mitochondrial OXPHOS [38]. PPAR agonists and antagonists may approach to modulate renal diseases like glomerulonephritis, glomerulosclerosis and diabetic nephropathy [39].

## 6. PPAR Signals in Heart

The heart consumes ATP to maintain its contractile function [40] and FAs are the main source of energy [41]. Around 70% of ATP used by the heart is obtained from FAO. Cardiac FAO is regulated at different stages such as FA uptake, triglyceride formation and storage, triglyceride lipolysis to release unesterified FAs, transfer of FA into mitochondria for FAO, and ATP production. Most of the proteins are transcriptionally regulated by PPARα [42]. PPARβ/δ in the myocardium controls glucose and lipid utilization, and promotes insulin sensitivity. The activity of PPARs in the heart is regulated by PGC-1α, which is responsible for mitochondrial biogenesis and metabolism [43]. 

PGC-1α is a highly expressed gene in the heart. PGC-1α interacts with PPARα, PPARγ, ERR, the retinoid X receptor, and nuclear respiratory factors to co-activate the transcription factors. Overexpression of PGC-1α significantly increases nuclear- and mitochondrial-related gene expression that changes the metabolic energy substrate from glucose to FA. The G-protein-coupled receptor kinase interacting protein-1 (GIT1) is a regulator of cardiac mitochondrial biogenesis that helps PGC-1α-regulated gene expression [44]. Under mild stress conditions like exercise, the level of FAO is increased due to oxidation of palmitate. However, the ATP produced by FAO is more than that produced by glucose oxidation because glucose oxidation needs oxygen, thus making it an efficient mode of cardiac energy production [45].

PPARα regulates cardiac FAO by activating genes in FA metabolism pathways such as FA uptake and β-oxidation, but not in the TCA cycle. Mitochondrial OXPHOS genes regulated by PGC-1α and ERRs in the heart are suppressed by the activation of PPARα, and thus, PPARα reduces glucose import and glycolysis by inducing cellular FA uptake and β-oxidation. Moreover, the importance of PPARα in regulating FAO was confirmed when PPARα knockout showed reduced FA uptake and β-oxidation. In addition, overexpression of PPARβ/δ induces FAO by upregulating genes in mitochondrial FA transport and β-oxidation. However, PPARβ/δ overexpression does not cause lipid accumulation and cardiac dysfunction. This may be due to high glucose utilization. The deletion of PPARβ/δ downregulates FAO genes and causes cardiac hypertrophy by lipid accumulation [1].

Patients with metabolic syndrome and aortic stenosis express high level of PPARγ in heart which is strongly correlated cardiac lipid accumulation and poor cardiac function. When the level of PPARγ is high under certain pathological conditions, it may cause cardiomyopathy [8]. 

## 7. PPAR Signals in Brain

All three PPARs (α, β/δ and γ) are expressed in the central nervous system (CNS) [46,47]. Among them, PPARγ is a key neuronal isoform used to regulate energy homeostasis [47,48,49,50,51,52]. It regulates genes involved in FA metabolism like acetyl-coenzyme A carboxylase (ACC), fatty acid synthase (FAS), and CPT1. It is expressed within the ventromedial nucleus (VMN) and the arcuate nucleus of the hypothalamus (ARC) of the brain. Overexpression of central PPARγ increases food intake, abdominal fat, activity of neuropeptide Y (NPY), and the expression of pro-opiomelanocortin (POMC) in the ARC. Conversely, the roles of PPARα and PPARβ/δ in energy metabolism are less understood. Knockdown of PPARβ/δ showed a decrease in leptin sensitivity with no change in food intake, but an increase in the expression of genes that are responsible for lipid uptake, lipid synthesis, and FA oxidation in the hypothalamus [51].

Recent studies suggest that activation of PPARα and/or PPARγ contribute to weight gain and obesity. Knockout of PPARγ in neurons and the hypothalamus prevents the development of diet-induced obesity (DIO). PPARα activation in the hypothalamus corrected the hypophagic phenotype in a model of increased CNS fatty acid sensing. Studies using rodent models suggest that the hypothalamic lipid accumulation is associated with obesity, and this may be due to the role of PPARβ/δ in the regulation of genes coded for lipid oxidation in the CNS [47]. The identification of PPARγ expression in dopaminergic neurons of the ventral tegmental area of the brain has helped to investigate a surprising role between food and other stimuli. ARC neurons, such as NPY/AgRP and POMC neurons with nuclear PPARγ, play important roles in the sensing of signals related to nutritional state, such as leptin, insulin, ghrelin, glucose, and FAs and transduce these signals to affect food intake, energy expenditure, and insulin sensitivity [53]. Thus, the maintenance of glucose homeostasis and food intake is controlled by central signaling of glucose, regulated by PPARs [51].

PPARγ agonist have shown their effect in Parkinson disease, Alzheimer disease, brain injury and amyotrophic lateral sclerosis. They are effective in suppressing the development of animal models of CNS inflammatory and neurodegenerative disorders [6]. 

## 8. PPAR Signals in Pancreatic β-Cells

PPARβ/δ is abundantly expressed in the pancreatic tissue of rats and human. PPARβ/δ is highly expressed in β-cells, but PPAR-α and -γ are relatively lowly expressed here [54,55,56,57]. PPARγ reduction leads to abnormal glucose metabolism in islets, meaning that it is required to maintain glucose metabolism [56]. PPARα and PPARγ play important roles in FA metabolism by regulating genes in FAO and energy uncoupling in mitochondria, such as CPT1 and UCP2. PPARβ/δ regulates mitochondrial energy metabolism and insulin secretion in β-cells [54,55,58], and increases the activation of FA β-oxidation enzyme genes, long chain acyl-CoA dehydrogenase (LCAD), PDK4, and UCP2. PPARβ/δ upregulates the mRNA level of PDK4 and increases the utilization of FAs, thus reducing insulin secretion. UCP2 is the bridge between mitochondrial energy metabolism and insulin secretion function [54]. The treatment of db/db mice with a PPARβ/δ agonist decreased blood glucose levels and improved insulin sensitivity and pancreatic islet function. It suggests that PPARβ/δ contributes as a FA sensor and to improve insulin secretion in β-cells [59]. Recent studies have shown that PPARα is ectopically expressed in INS-1 cells that could induce lipid accumulation alone with an increase in β-oxidation. PPARγ promotes FA disposal in pancreatic β-cells [56]. 

## 9. PPAR Signals in Intestine

PPARα and PPARβ/δ are highly expressed in the intestine [60,61]. In the lumen of the colon, short chain FAs (SCFAs) such as acetic acid, propionic acid, and butyric acid are produced. A recent study showed that propionate lowers FA content in the plasma and reduces food intake. Dietary triglyceride (TG) is hydrolyzed into free FAs in the lumen of the intestine. These free FAs are taken up by intestinal epithelial cells to the endoplasmic reticulum where they are resynthesized into TG. This intestinal TG metabolism process is very important for systemic energy homeostasis [61]. 

Animal studies have demonstrated relationships between intestinal colonization, energy utilization, and weight gain. The mechanism of this process involves regulation of angiopoietin-like protein 4 (ANGPTL4) expression in the intestinal epithelium. ANGPTL4 is a secreted protein that regulates lipid and glucose homeostasis. The amino terminal domain of ANGPTL4 inhibits lipoprotein lipase activity and decreases triglyceride uptake and storage. In addition, it induces lipolysis and results in the elevation of circulating triglyceride levels. Deletion of ANGPTL4 results in changes in metabolism, decreased intestinal absorption of oils, and thickening of the intestinal mucosa. PPARγ is involved in regulating FA metabolism through β-oxidation. PPARγ regulates ANGPTL4 expression and PPRE within the third intron of the ANGPTL4 gene. SCFAs activate PPARγ and are the products of dietary fibers and main energy sources for colonocytes [62]. 

PPARα agonist Wy-14643 induces the protein expression of enzymes involved in FAO and ketogenesis such as CPT1A and mitochondrial 3-hydroxy-3-methylglutaryl-CoA synthase in the small intestine [63]. PPARα regulates various transporters and phase I enzymes involved in FA uptake and oxidation. Nutritional-activated PPARα controls FAO and cholesterol and glucose transport [64]. During fasting, PPARα plays an important role in regulating transporter and phage I/II metabolism genes in the small intestine [65]. 

Similarly, administration of another PPARα modulator, K-877, regulates intestinal FAO and apolipoprotein mRNA expression and reduces plasma TG levels. K-877 administration significantly reduces Npc1l1 expression and increases Abca1 expression. Npc1l1 is a rate-limiting transporter for cholesterol absorption in the small intestine of mice, whereas Abca1 is an important molecule involved in HDL-C production by transporting intracellular cholesterol from the small intestine. Intestinal Abca1 deficiency leads to deficient HDL biogenesis and therefore reduces cholesterol influx in to the circulation [66]. 

## 10. Co-Regulators of PPAR in Energy Homeostasis

Balanced energy homeostasis is the result of high pathway interconnectivity and feedback control. The Nuclear Receptor Signaling Atlas has reported around 320 nuclear receptor co-regulators, and there have been 38 co-regulators identified for PPARs alone. Not only do PPARs contribute to systemic energy homeostasis on their own, but crosstalk of PPARs with various pathways also has an effect [67]. Co-activators and co-repressors collectively regulate mitochondrial energy balance. PPARγ and PGC-1α are the co-regulators for induction of mitochondrial oxidative metabolism. Nuclear co-repressor 1 (NCOR1) antagonizes the effect of PGC1α on mitochondria. Knocking out NCOR1 phenotypically mimics PGC-1α overexpression. PGC-1α participates in the transcriptional response of ERR and PPARs. Nuclear receptor interacting protein 1 (NRIP1) binds to the PPAR nuclear receptors, as well as ERR, and represses the expression of target genes that are involved in energy consumption. NIRP1-deficient mice are lean, and show increased insulin sensitivity and glucose tolerance, and resistance to diet-induced obesity [1,67,68]. Under different nutritional conditions, hepatocyte nuclear factor α (HNFα), Hes6, and the PPARs balance the expression of each other and regulate the transcription cascade in metabolism [23,69]. PPARγ with the transcription factor, CCAAT/enhancer-binding protein α (C/EBPα), is an important driver in the late stage of adipogenesis. Mice with the liver specific knockout of mediator complex subunit 1 (MED1) were shown to have impaired PPARα and PPARγ activities. This suggests that MED1 plays an important role in energy homeostasis via PPARs [67]. 

PPARs regulate lipid and glucose metabolism and are involved in a variety of diseases, ranging from metabolic disorder to cancer [9,70]. They have a significant, energetic, plastic, and signaling roles in the pathophysiology of cancer cells. Most cancer cells show increased aerobic glycolysis and use PPAR signaling pathways to generate ATP as a main source of energy. Stimulated peroxisomal β-oxidation increases free radical oxygen species that may increase oxidative stress. This significantly contributes to the carcinogenic properties of PPAR ligand in rodents, particularly in the liver. Activation of PPARs (α, β/δ, γ) by natural or synthetic agonists can inhibit growth and induce differentiation or death of tumor cells. Synthetic ligands of PPARs show an important link with cancer. PPARγ and PPARα ligands have been shown to promote the differentiation of various tumor cell lines, including breast, lung, prostate, leukemia, colon, melanoma, and liver cancers [71].

PPARs are involved in controlling the genes responsible for not only energy homeostasis but also cell proliferation, apoptosis, tumorigenesis, and metabolic disease development [72]. A previous study showed that ANGPTL4 and PPARs play potential synergistic roles in the crosstalk between metabolic syndromes and cancer [10]. PPAR transcriptional activity can be modulated through cross-talk with phosphates and kinases, including ERK1/2, P38-MAPK, PKC, AMPK, and GSK3. PPARs activate the transcription of genes involved in anticancer effects in a variety of human tumors. PPARγ appears to be mostly involved in tumorigenesis regulation [73]. The shortage of vitamin D and decreased level of PPARγ may be involved in obesity and cancer development [74]. PPARβ/δ is involved in the initiation and promotion of mammary tumorigenesis by regulating metabolism, inflammation, and immune tolerance [75]. All PPARs, including α, β/δ, and γ, have been shown to be important in lung cancer biology. PPARα activation inhibits tumorigenesis through its antiangiogenic and anti- inflammatory effects. Activated PPARγ is also anti-tumorigenic and anti-metastatic, regulating several function of cancer cells and controlling the tumor microenvironment [76]. Among the synthetic ligands of PPARγ, thiazolidinediones, which are used to treat diabetes mellitus type 2, increase the risk of bladder cancer [77]. FAs from conjugated linoleic acid-enriched egg yolks (EFA-CLA) act as a potential ligand for PPAR receptors in the breast cancer cell line MCF-7. PPAR-responsive genes can be regulated by EFA-CLA, leading to reduced tumor cell proliferation, which has a greater influence than non-enriched FAs or single synthetic CLA isomers [78]. PPAR modulators may have beneficial effects as chemo-preventive agents. However, it remains unclear whether PPARs act as oncogenes or tumor suppressors [9]. The co-regulators of PPARs in carcinogenic process is summarized in Figure A2. Further studies are needed to develop new approaches for treating neoplasia. 

PPARs are involved in various pathways for energy homeostasis in different organs. These pathways are affected in disease conditions and cause the metabolic energy imbalance. Thus, PPARs can provide therapeutic targets for different diseases such as dyslipidemia, diabetes, obesity, inflammation, neurodegenerative disorders and cardiomyopathy [6,8].

## 11. Conclusions

**Figure A1:**
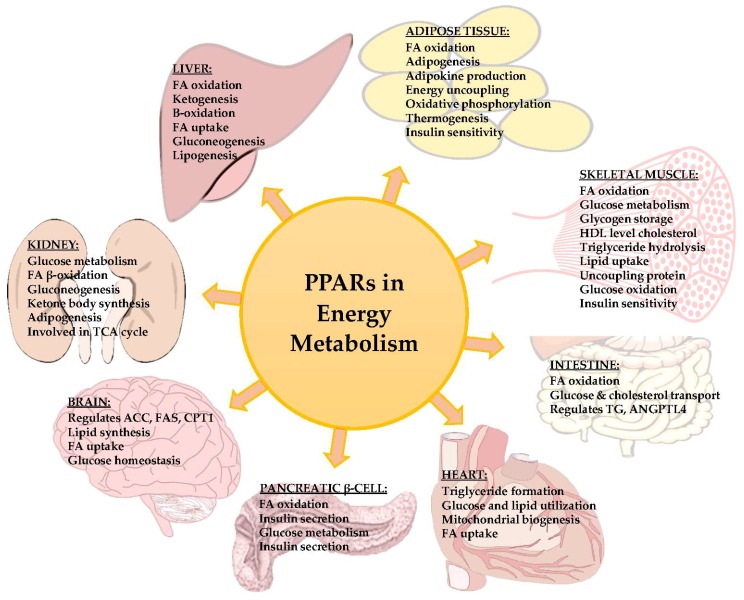
A schematic overview of role of PPARs in energy metabolism in various body organs.

**Figure A2:**
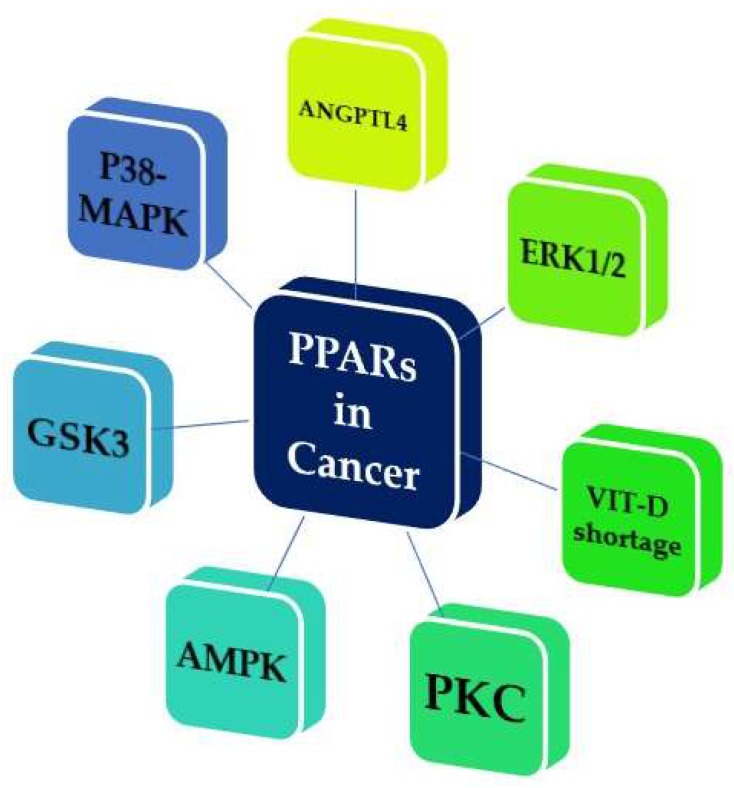
Co-regulators of PPARs in carcinogenic process.

Thus, PPARs are crucial transcriptional factors involved in energy metabolism for the whole body and the three isotypes have complementary and distinct metabolic activities. PPARs also act with other co-regulators in the maintenance of energy homeostasis. The overview of PPARs in cellular and whole body energy homeostasis is illustrated in Figure A1. However, the exact molecular mechanism of PPARs within energy metabolism remains unclear. Future research in this field should be oriented towards the molecular mechanism, to ensure the use of PPAR as a therapeutic targets. 

## Abbreviations

PPARs	Peroxisome proliferator-activated receptors
FAs	Fatty acids
PPREs	PPAR response elements
FAO	Fatty acid oxidation
CPT1	Carnitine palmitoyltransferase 1
HDL	High-density lipoprotein
WAT	White adipose tissue
BAT	Brown adipose tissue
GPDH	Glycerol-3-phosphate dehydrogenase
OXPHOS	Oxidative phosphorylation
PDK4	Pyruvate dehydrogenase kinase 4
LDHB	Lactate dehydrogenase B
PGC-1α	PPARγ coactivator-1α
UCP1	Uncoupling protein 1
ATP	Adenosine triphosphate
TFEB	Transcription factor EB
TCA	Tricarboxylic acid
GIT1	G-protein-coupled receptor kinase interacting protein-1
ACC	Acetyl-coenzyme A carboxylase
FAS	Fatty acid synthase
VMN	Ventromedial nucleus
ARC	Arcuate nucleus of the hypothalamus
NPY	Neuropeptide Y
POMC	Pro-opiomelanocortin
CNS	Central nervous system
DIO	Diet induced obesity
LCAD	Long chain acyl-CoA dehydrogenase
NCOR1	Nuclear co-repressor 1
HNFα	Hepatocyte nuclear factor α
C/EBPα	CCAAT/enhancer-binding protein α
MED1	Mediator complex subunit 1
ANGPTL4	Angiopoietin-like protein 4
TG	Triglyceride
HMG-CoAS2	Mitochondrial 3-hydroxy-3-methylglutaryl-CoA synthase
SCFAs	Short chain fatty acids
EFA-CLA	Fatty acids from conjugated linoleic acid-enriched egg yolks
CLA	Conjugated linoleic acid
NAFLD	Non-alcoholic fatty liver disease
NASH	Non-alcoholic steatohepatitis
TZD	Thiazolidinediones
ERK1/2	Extracellular signal-regulated kinase type 1 and 2
P38-MAPK	Mitogen-activated protein kinase p38
PKC	Protein kinase C
AMPK	5’Adenosine monophosphate-activated protein kinase
GSK3	Glycogen synthase kinase 3
